# Identification and characterization of two linear epitope motifs in hepatitis E virus ORF2 protein

**DOI:** 10.1371/journal.pone.0184947

**Published:** 2017-09-28

**Authors:** Heng Wang, Weidong Zhang, Honglang Gu, Wanli Chen, Meng Zeng, Chihai Ji, Ruyue Song, Guihong Zhang

**Affiliations:** 1 College of Veterinary Medicine, South China Agricultural University, Guangzhou, Guangdong Province, People’s Republic of China; 2 Guangdong Provincial Key Laboratory of Prevention and Control for Severe Clinical Animal Diseases, Guangzhou, Guangdong Province, People’s Republic of China; 3 Hospital of South China Agricultural University, Guangzhou, Guangdong Province, People’s Republic of China; 4 MOA Key Laboratory of Animal Vaccine Development, Guangzhou, Guangdong Province, People’s Republic of China; Centers for Disease Control and Prevention, UNITED STATES

## Abstract

Hepatitis E virus (HEV) is responsible for hepatitis E, which represents a global public health problem. HEV genotypes 3 and 4 are reported to be zoonotic, and animals are monitored for HEV infection in the interests of public hygiene and food safety. The development of novel diagnostic methods and vaccines for HEV in humans is thus important topics of research. Opening reading frame (ORF) 2 of HEV includes both linear and conformational epitopes and is regarded as the primary candidate for vaccines and diagnostic tests. We investigated the precise location of the HEV epitopes in the ORF2 protein. We prepared four monoclonal antibodies (mAbs) against genotype 4 ORF2 protein and identified two linear epitopes, G438IVIPHD444 and Y457DNQH461, corresponding to two of these mAbs using phage display biopanning technology. Both these epitopes were speculated to be universal to genotypes 1, 2, 3, 4, and avian HEVs. We also used two 12-mer fragments of ORF2 protein including these two epitopes to develop a peptide-based enzyme-linked immunosorbent assay (ELISA) to detect HEV in serum. This assay demonstrated good specificity but low sensitivity compared with the commercial method, indicating that these two epitopes could serve as potential candidate targets for diagnosis. Overall, these results further our understanding of the epitope distribution of HEV ORF2, and provide important information for the development of peptide-based immunodiagnostic tests to detect HEV in serum.

## Introduction

Hepatitis E virus (HEV) causes hepatitis E, which is considered to be a significant public health problem. It spreads in both developing [1] and industrialized countries [2], and is responsible for around 20 million infections and 70 thousand deaths each year according to World Health Organization. HEV is a single-stranded, positive-sense RNA virus classified into the genus *Orthohepevirus*, family *Hepeviridae* [3]. More than half of acute viral cases have been reported to be caused by HEV in endemic regions, and the mortality of pregnant women infected with HEV who progress to fulminant hepatitis is as high as 25% in developing countries [4]. HEV was originally classified into four genotypes, but is now classified into seven genotypes [3], including genotypes 1–4 and 7, which can infect humans [5]; genotypes 1 and 2 are restricted to humans, while genotypes 3 and 4 have expanded natural host ranges with a zoonotic characteristic, and genotype 7 is mainly derived from camels. In addition, avian HEV belongs to an independent species of *Orthohepevirus B*, which shares nearly 50% nucleotide identity and similar antigen epitope characteristics in open reading frame (ORF) 2 protein with mammalian HEV isolates [6,7]. The HEV genome includes three partially overlapping ORFs. ORF1 encodes a non-structural protein that participates in the process of virus replication, while ORF3 encodes a small multifunctional protein. ORF2 encodes the major HEV structural protein, the capsid protein, which includes three domains designated S, M, and P [8]. Previous studies indicated that ORF2 protein includes both linear and conformational epitopes, including important neutralizing epitopes on the P domain [9,10]. ORF2 protein is therefore considered to be the ideal candidate for the development of vaccines and diagnostic tests. However, the precise location of the epitopes on ORF2 protein remains unclear.

In this study, we prepared four monoclonal antibodies (mAbs) against ORF2 protein with the aim of identifying the precise location of the epitopes using a phage display biopanning method. The results will improve our understanding of the epitope distribution of HEV ORF2 and further the development of effective diagnostic tests for HEV.

## Materials and methods

### Preparation of specific mAbs

The recombinant plasmid, pET32a-ORF2, containing the truncated ORF2 gene (nucleotides 1240–2022) of HEV genotype 4 derived from swine (GenBank: JX855794, constructed and maintained in our laboratory) was transformed into *Escherichia coli* strain BL21 (DE3) pLysS (Invitrogen, CA, USA). ORF2 expression was induced with isopropyl-β-D-thiogalactoside at a final concentration of 1 mM at 37°C. Bacterially expressed protein was identified by sodium dodecyl sulfate-polyacrylamide gel electrophoresis (SDS-PAGE). ORF2 protein in inclusion bodies was purified using the gel-cutting method [11] and purified proteins were analyzed by SDS-PAGE and western blotting.

Purified recombinant ORF2 protein was used as an antigen to immunize six 5-week old Balb/c mice, and mAbs were prepared using standard methodology [12]. All of the Balb/c mice were kept at 21 to 25°C on a 12 h light/dark cycle and were given free access to food and water. Body weight of the mice was measured weekly and mice with high serum titers were euthanized by CO_2_ inhalation. All animal experiments were performed according to the Guide for the Care and Use of Laboratory Animals of the Ministry of Science and Technology of the People’s Republic of China. This animal study was also approved by the Animal Experimental Ethics Committee of South China Agricultural University. The specificity of these mAbs for ORF2 protein was determined by ELISA. Briefly, purified recombinant ORF2-His fusion protein and 6×histidine tag (6×His-tag) protein (1 μg/well diluted in 0.1 M NaHCO_3_, pH 8.6), respectively, were coated onto ELISA plate wells overnight at 4°C. Polyclonal antibody against ORF2 was used as a positive control and normal mouse serum as a negative control. Subsequent steps were conducted as described previously [12].

The prepared mAbs were also used to detect HEV ORF2 protein expressed in baby hamster kidney (BHK) cells transfected with pcDNA3.1-ORF2 (nucleotides 1240–2022) plasmids, by immunofluorescence assay (IFA). The detailed procedures were performed as described previously [12].

### Phage display biopanning on HEV ORF2 mAbs

The purified ORF2 mAbs were applied as targets and subjected to biopanning using a Ph.D.-12 Phage Display Peptide Library Kit (New England Biolabs, MA, USA). The procedure was performed according to the manufacturer’s instructions and a previous study [13]. The ratio in each round was calculated to analyze the enrichment efficiency using the titer of the amplified phages in the input buffer (input value) and in the elution buffer (output value).

Individual phage clones were selected from the final round and amplified in *E*. *coli* ER2738 (New England Biolabs, USA), followed by precipitation according to the manufacturer’s protocols. Genes encoding the exogenous peptides of M13 were amplified by polymerase chain reaction (PCR) using the forward primer 5'-TCACCTCGAAAGCAAGCTGA and reverse primer 5'-CCCTCATAGTTAGCGTAACG. The PCR parameters were: 95°C for 5 min, 30 cycles of 95°C for 30 s, 56.5°C for 30 s, 72°C for 30 s, with a final extension at 72°C for 5 min.

The binding activities of phage clones displaying different high frequency peptide sequences, at concentrations of 10^10^–10^4^ plaque-forming units (pfu) diluted in 0.1 M NaHCO_3_ (pH 8.6), to ORF2 mAbs were detected by ELISA, according to the instruction of Ph.D.-12 Phage Display Peptide Library Kit.

### Sequence analysis and spatial location prediction

The deduced encoding amino acid sequences were analyzed by post-sequencing and compared with swine HEV genotype 4 ORF2 protein (GenBank: JX855794). Conservation of the newly identified epitopes among various types of HEVs, including genotypes 1, 2, 3, 4, and avian HEV, was analyzed using the MegAlign module of DNASTAR software (Madison, WI, USA).

The molecular structure of HEV ORF2 protein based on its amino acid sequence was predicted using I-TASSER (http://zhanglab.ccmb.med.umich.edu/I-TASSER/) [14,15]. Rasmol 2.7.5 (www.RasMol.org) and Swiss-Pdb Viewer 3.7 (http://spdbv.vital-it.ch/) were applied to highlight areas of interest on the ORF2 protein.

### Peptide synthesis and affinity of peptides to ORF2 mAbs

The candidate peptides from the phage display biopanning were synthesized by ChinaPeptides Co., Ltd. (Shanghai, China), according to the standard used in our previous study [13]. The affinity of the synthetic peptides to ORF2 mAbs was detected by ELISA. Briefly, peptides (100 μl/well) at a concentration of 10 μg/ml diluted in 0.1 M NaHCO_3_ buffer (pH 8.6) were coated on 96-well plates overnight at 4°C. After blocking with TBSB (50 mM Tris-HCl, pH7.5, 150 mM NaCl, 1% bovine serum albumin), four prepared ORF2 mAbs were added into the plate, respectively, at a concentration of 1 μg/ml. Normal mouse serum was used as a negative control. Horseradish peroxidase-conjugated anti-mouse IgG antibody was added and the color was developed using 3,3’,5,5’-Tetramethylbenzidine. The value of optical density 450 (OD_450_) was detected by an ELISA plate reader.

### Peptide-based ELISA to detect anti-HEV-positive serum

Two candidate peptides were used to detect anti-HEV-positive serum by ELISA. One hundred positive and 86 negative sera collected from the field were confirmed using a commercial ELISA kit (Wantai Biological Pharmacy Co., Beijing, China) and maintained in our laboratory. Candidate peptides (10 μg/well diluted in 0.1 M NaHCO3, pH 8.6) were coated onto ELISA plates overnight at 4°C. Subsequent steps were conducted as described previously [12]. (OD_450_ Sample—OD_450_ Blank)/ (OD_450_ Negative control—OD_450_ Blank) > 2 was determined as positive result. The ratio < 1.5 was considered as negative, meanwhile, the ratio ≤ 2 and ≥ 1.5 was regarded as suspected one.

### Statistical analyses

All measurements were repeated in triplicate and mean values ± standard deviation were calculated using GraphPad Prism software (version 5.00; GraphPad Software, San Diego, CA, USA). Statistical analysis was conducted by one-way analysis of variance, and *P* values <0.05 were considered statistically significant.

## Results

### Generation and identification of anti-ORF2 protein mAbs

**Fig 1:**
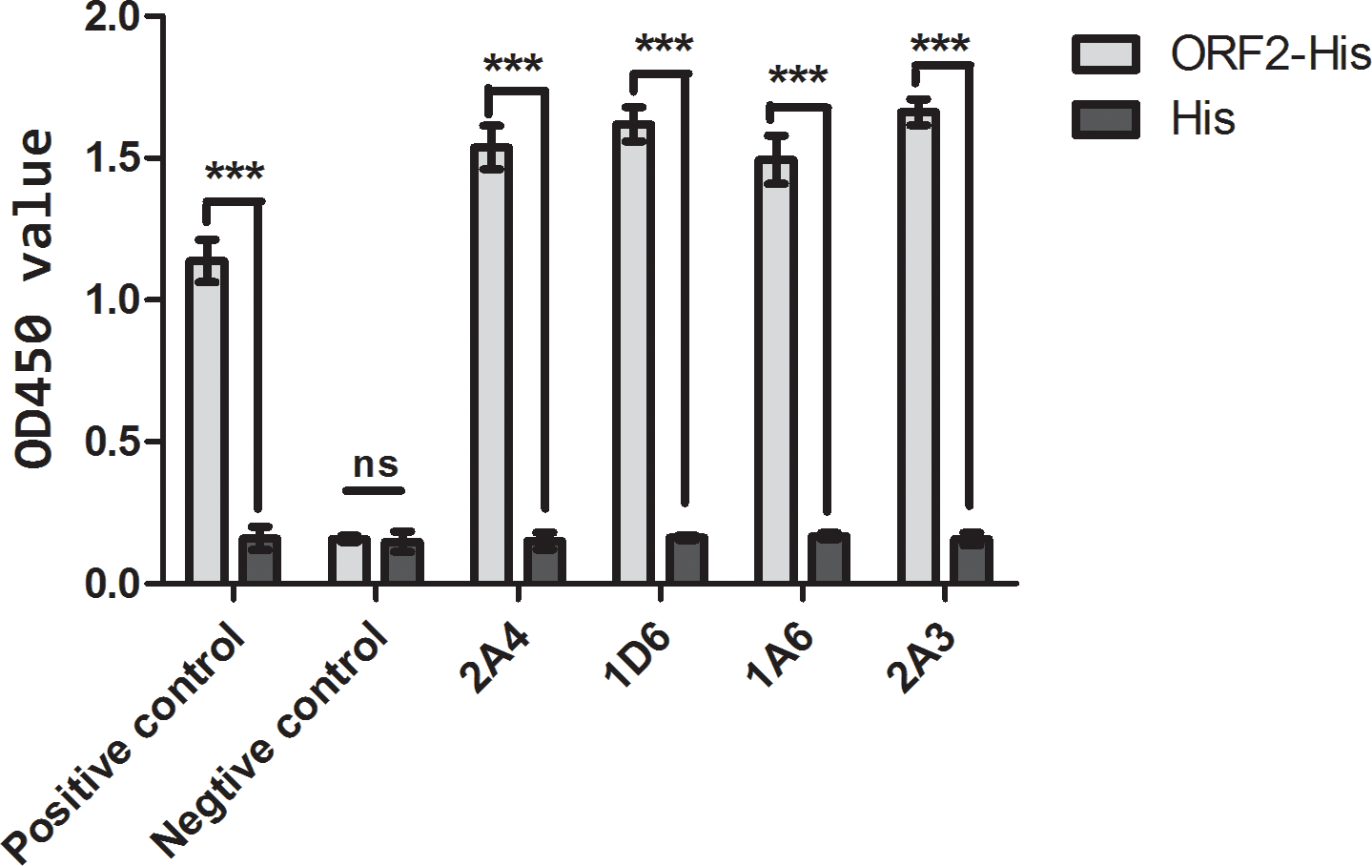
ELISA to determine specificity of prepared mAbs for ORF2 protein. ***P<0.001; nc, *P*>0.05.

Bacterially expressed HEV ORF2 recombinant protein fused to a His-tag was purified from gels and used to immunize BALB/c mice. Recombinant ORF2 proteins were used to screen positive mAbs produced by hybridomas, and the 6×His-tag recombinant protein was used to eliminate the possibility of mAbs reacting with the His-tag protein. Four mAbs, 2A4, 1D6, 1A6, and 2A3, were identified by ELISA, all of which reacted specifically with His-tagged ORF2 protein and not His-tag protein (Fig 1).

**Fig 2:**
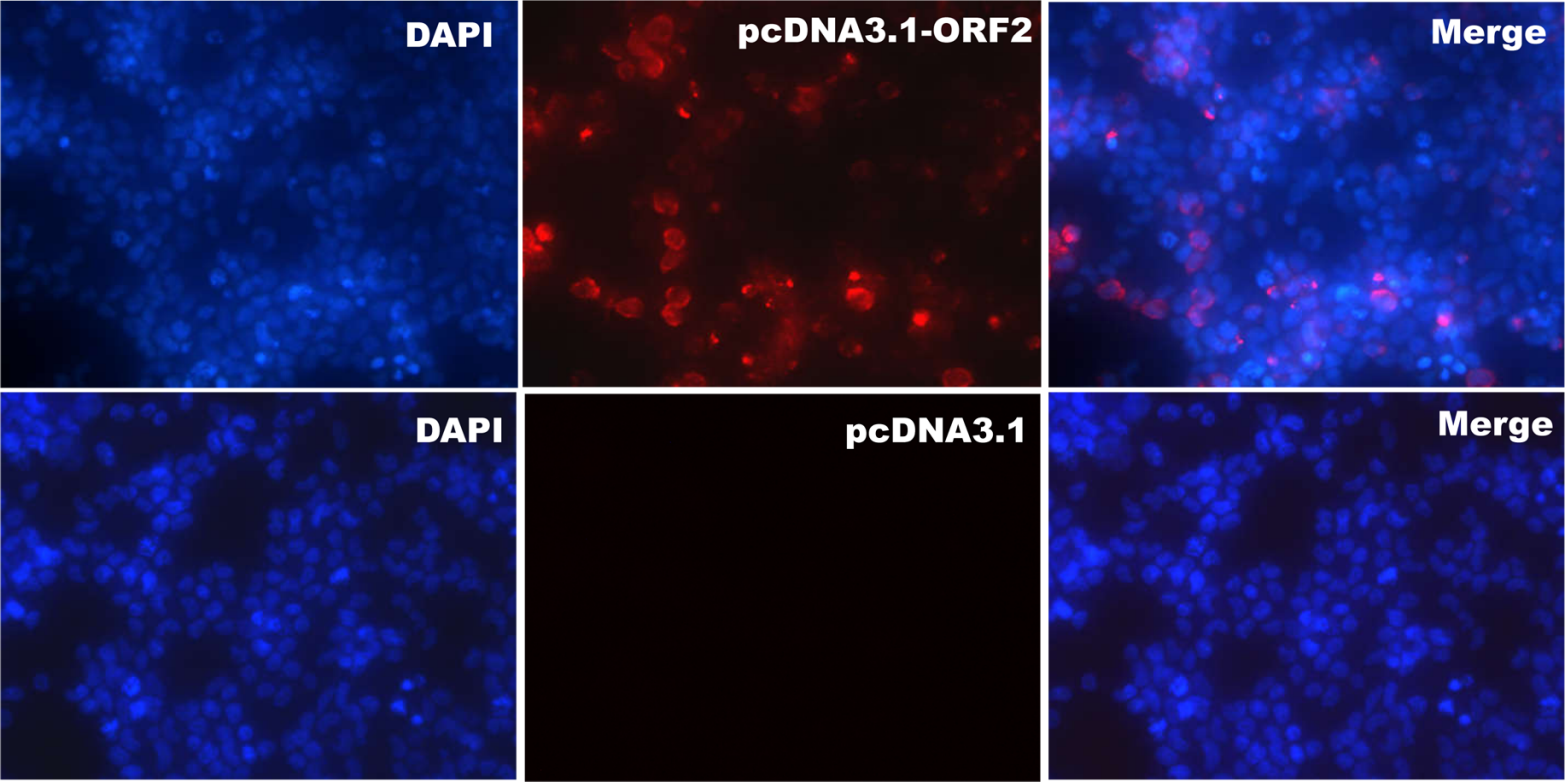
HEV ORF2 protein expression in BHK cells transfected with pcDNA3.1-ORF2 by mAb 2A4. Nuclei were stained with 4',6'-diamidino-2-phenyl-indole, which revealed blue fluorescence. Red fluorescence indicated a positive signal. BHK cells transfected with pcDNA3.1 were designated as the control. The results with the other three mAbs were similar (S1 Fig).

The specificity of these mAbs for identifying HEV ORF2 protein expressed *in vitro* was confirmed by IFA. A red fluorescence signal was detected in the cytoplasm of BHK cells transfected with pcDNA3.1-ORF2 recombinant plasmids and each of the four prepared mAbs. In contrast, no red signal was observed in BHK cells transfected with pcDNA3.1 empty vector (Fig 2).

### Biopanning to screen phages displaying mAb-specific peptides

The four mAbs were used as immobilized targets in ELISA plate wells, and four rounds of biopanning were performed to identify the specific phages. Dynamic changes in enrichment efficacy for each round showed that the phage titers in the elution buffer increased with increasing number of panning rounds (S1 Table). Thirty phage clones for each mAb were selected randomly from the last round of biopanning, amplified in *E*. *coli* ER2738, and the DNA genomes were extracted from these phages. Heterologous genes encoding the peptides displayed on the phages were amplified by PCR using specific primers, and the deduced peptide sequences were analyzed and classified. Most of the selected clones were positive by PCR, but some lacked exogenous sequences. Sequence alignment using DNASTAR software revealed four, seven, eight, and six kinds of consensus peptide sequences comprising 12 amino acids displayed on the phages to mAbs 2A4, 1D6, 1A6, and 2A3 respectively (Table 1).

**Table 1:** Deduced amino acid sequences of peptides displayed on selected phages.

mAb	12-mer amino acid sequence	Clone no.	Total clone no.
2A4	GFDTQHTNMIES	11	26
	VGFDTQHVQFYP	8	
	AGFDAQHVCSLS	5	
	ASYDNQHHSYIN	1	
1D6	WTIGLDSPYDTL	5	21
	DLFRSLYLDTLT	3	
	STFGLLSPPHLL	3	
	SSVGLLSPYPDL	3	
	YGDNRGLLVPYE	3	
	STLGLHHPYEAE	2	
	SVDNLYDNQHTG	2	
1A6	AIRVPHDFIVSP	6	27
	NFITVPNDSHTM	6	
	RILIPNDYYPIQ	3	
	GILIPHDNRNNP	3	
	QIVIPRDTFSDN	2	
	NAVPSSIRIPRD	2	
	SVTSGIRITDDS	2	
	ESIHVPKDHQYR	2	
2A3	SIGLRAPYERPV	16	23
	WLPNELLLRLSS	2	
	VMPNFADLGLIT	2	
	MPGLREPYEYSM	1	
	GDFITALNRELQ	1	
	SENLRWWRNLEW	1	

All the consensus peptide sequences were compared with the ORF2 protein. Multiple sequence alignment analysis revealed strong homology among the peptide sequences displayed on the phages to mAbs 2A4 and 1A6 (Tables 2 and 3). One consensus peptide sequence displayed on the phages to mAb 1D6, SVDNLYDNQHTG (C1), also showed high homology to ORF2 protein and the peptide sequences displayed on the phages to mAb 2A4 (Table 3).

**Table 2:** Alignment of peptide sequences displayed on selected phages corresponding to mAb 2A4.

Sequence	Amino acid sequence												
A1		G	**F**	**D**	T	**Q**	**H**	T	**N**	M	I	E	S
A2	V	G	**F**	**D**	T	**Q**	**H**	V	**Q**	F	Y	**P**	
A3	A	G	**F**	**D**	A	**Q**	**H**	V	C	S	L	S	
A4	A	S	**Y**	**D**	**N**	**Q**	**H**	H	S	Y	I	N	
HEV ORF2	Q	D	**Y**	**D**	**N**	**Q**	**H**	E	**Q**	D	R	**P**	T

Bold type indicates continuous consensus amino acid sequence displayed on the selected phages coincident with HEV ORF2 protein. Underlined bold type indicates amino acid in peptide sequence displayed on the selected phages with same classification as corresponding amino acid in HEV ORF2 protein.

**Table 3:** Alignment of peptides sequences displayed on selected phages corresponding to mAb 1A6.

Sequence name	Amino acid sequence																
B1						A	**I**	R	**V**	**P**	**H**	**D**	F	I	V	S	P
B2					N	F	**I**	T	**V**	**P**	N	**D**	S	H	T	M	
B3						R	**I**	**L**	**I**	**P**	N	**D**	Y	Y	P	I	Q
B4						**G**	**I**	**L**	**I**	**P**	**H**	**D**	N	R	N	N	P
B5						Q	**I**	**V**	**I**	**P**	R	**D**	T	F	S	D	N
B6	N	A	V	P	S	S	**I**	R	**I**	**P**	I	**D**					
B7		S	V	T	S	**G**	**I**	R	**I**	T	D	**D**	S				
B8					E	S	**I**	H	**V**	**P**	K	**D**	H	Q	Y	R	
HEV ORF2	A	Q	Q	D	K	**G**	**I**	**V**	**I**	**P**	**H**	**D**	I	D	L	G	E

Bold type indicates continuous consensus amino acid sequence displayed on the selected phages coincident with HEV ORF2 protein. Underlined bold type indicates amino acid in peptide sequence displayed on the selected phages with same classification as corresponding amino acid in HEV ORF2 protein.

Further analysis indicated that the four consensus peptide sequences displayed on the phages to mAb 2A4 contained a homology sequence YD#DH (# represented amino acid T or A), which was analogous to the sequence D458NQH461 of HEV ORF2 protein. Sequence A4 had a five-amino acid sequence, YDNQH, coincident with the sequence Y457DNQH461 of ORF2 protein (Table 2). Similarly, the sequence C1 contained the same five-amino acid sequence (Table 4).

**Table 4:** Alignment of peptide sequences displayed on selected phages corresponding to mAb 1D6.

Sequence name	Amino acid sequence											
C1	S	**V**	D	**N**	L	**Y**	**D**	**N**	**Q**	**H**	H	S
HEV ORF2	V	**V**	I	**Q**	D	**Y**	**D**	**N**	**Q**	**H**	E	Q

Bold type indicates continuous consensus amino acid sequence displayed on the selected phages coincident with HEV ORF2 protein. Underlined bold type indicates amino acid in peptide sequence displayed on the selected phages with same classification as corresponding amino acid in HEV ORF2 protein.

Sequence alignment revealed that the eight kinds of consensus peptide sequences displayed on the phages to mAb 1A6 also contained a sequence homologous with G438IVIPHD444 of HEV ORF2 protein. The sequence B4 contained a seven-amino acid sequence, GILIPHD, analogous to the sequence G438IVIPHD444 (Table 3), given that amino acids L and I are both branched chain amino acids with similar characteristics and structures.

In contrast, no homologous sequences, except for the sequence C1, were found for the peptide sequences displayed on the phages to mAb 2A3 and 1D6.

### Binding activity of selected phage clones

**Fig 3:**
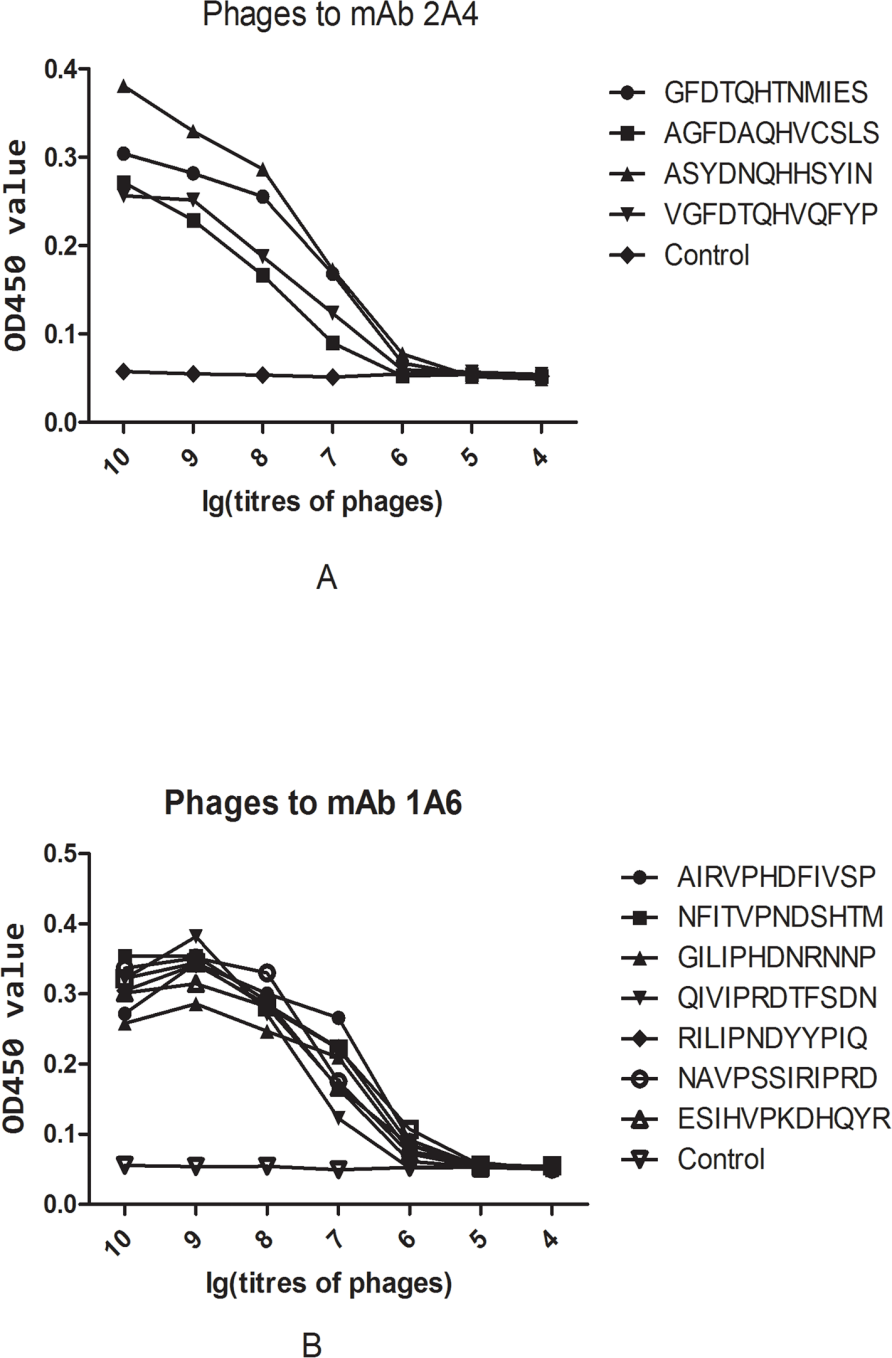
Binding analysis of selected phages to mAbs 2A4 and 1A6. (A) ELISA of selected phages to corresponding mAb 2A4. (B) ELISA of selected phages to corresponding mAb 1A6. Original mixed phage library was used as control. OD_450_ values of the individual phages and control are shown on the *y* axis. Log values of phage titers are shown on the *x* axis.

According to the above results, the phages to mAbs 2A4 and 1A6, which displayed strongly homologous consensus peptide sequences, were amplified and serially diluted and their binding activities to the respective mAbs were confirmed by ELISA (Fig 3). All the selected phages at high concentrations (10^7^–10^10^ pfu) showed strong binding to their respective mAbs, compared with the original mixed phage library designated as the control (*P*<0.001). There was no significant difference in binding activity among these positive phages at any given concentration (*P*>0.05). The binding activities of each phage decreased in a concentration-dependent manner, with no significant difference in binding activities to their respective mAbs compared with the control group at low concentrations (10^4^–10^5^ pfu) (*P*>0.05).

### Specific sequence alignment among different types of HEV ORF2 protein

**Fig 4:**
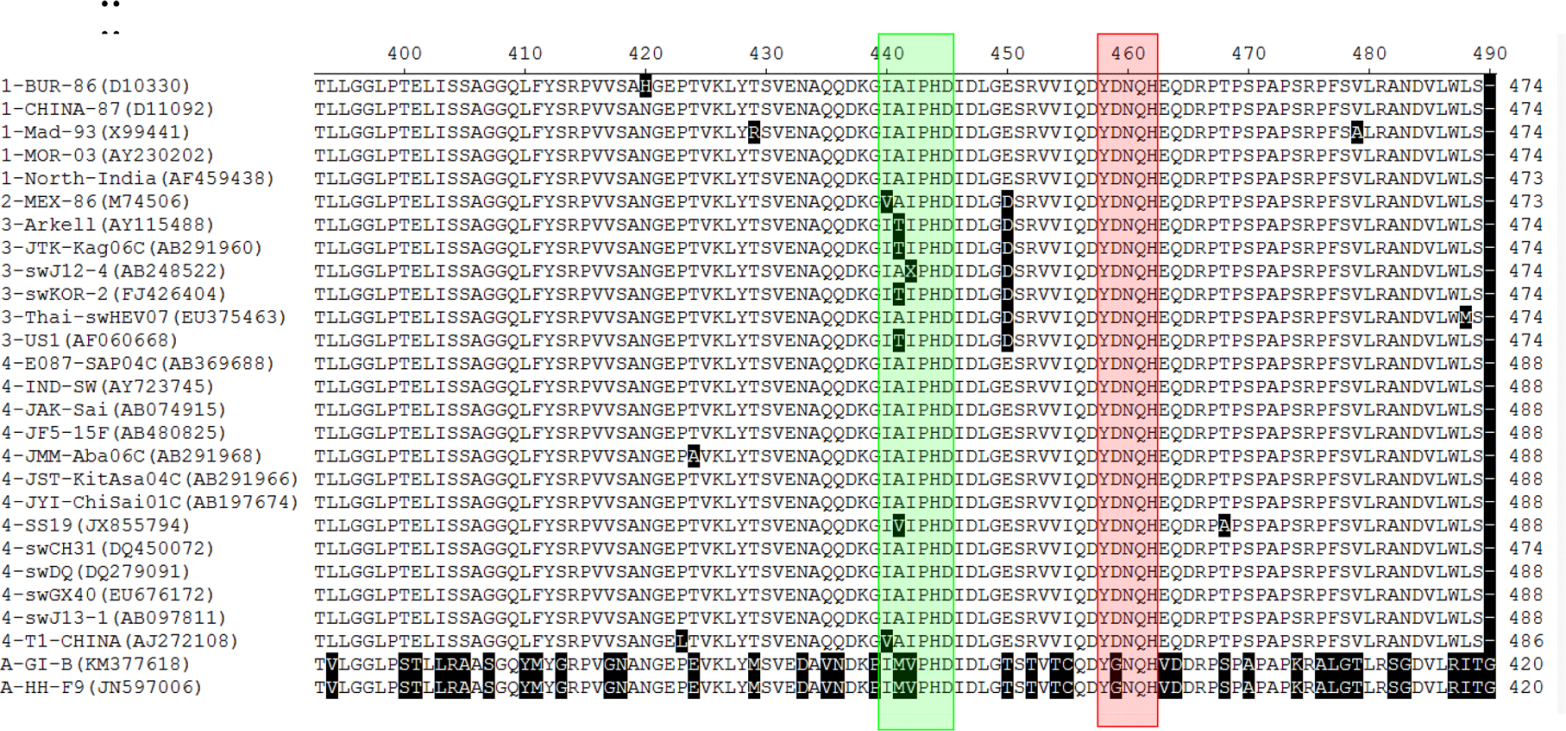
Conservation of newly identified epitopes among genotypes 1, 2, 3, 4, and avian HEV. The IVIPHD epitope motif is indicated in a green box and YDNQH is indicated in a red box. Strain names for each sequence are shown at left side; the first number or letter represents genotype 1–4 or avian genotype, respectively. Numbers in brackets represent GenBank accession numbers of the selected HEV strains. Residues shaded in black represent those that differ from most strains.

Based on the aforementioned results, we hypothesized that the sequences G438IVIPHD444 and Y457DNQH461 might be linear epitopes of HEV genotype 4 ORF2 protein. We further analyzed the conservation of these two suspected epitopes among various types of HEVs in a panel of 27 HEV ORF2 sequences from genotypes 1, 2, 3, 4, and avian isolates selected from GenBank. Multiple sequence alignment analysis indicated that the suspected epitope Y457DNQH461 was highly conserved among all the selected isolates, including avian HEVs. There was no mutation in this motif among HEV genotypes 1, 2, 3, and 4, and only one D to G mutation compared with avian HEVs (Fig 4). The other suspected epitope G438IVIPHD444 was also highly conserved, but there was a minor mutation in this motif among some isolates. In contrast with most HEVs, there was a two-consecutive amino acid mutation, AI to MV, in avian HEVs (Fig 4). In summary, these two suspected linear epitopes may be universal to genotypes 1, 2, 3, 4, and avian HEVs.

### Structure prediction

**Fig 5:**
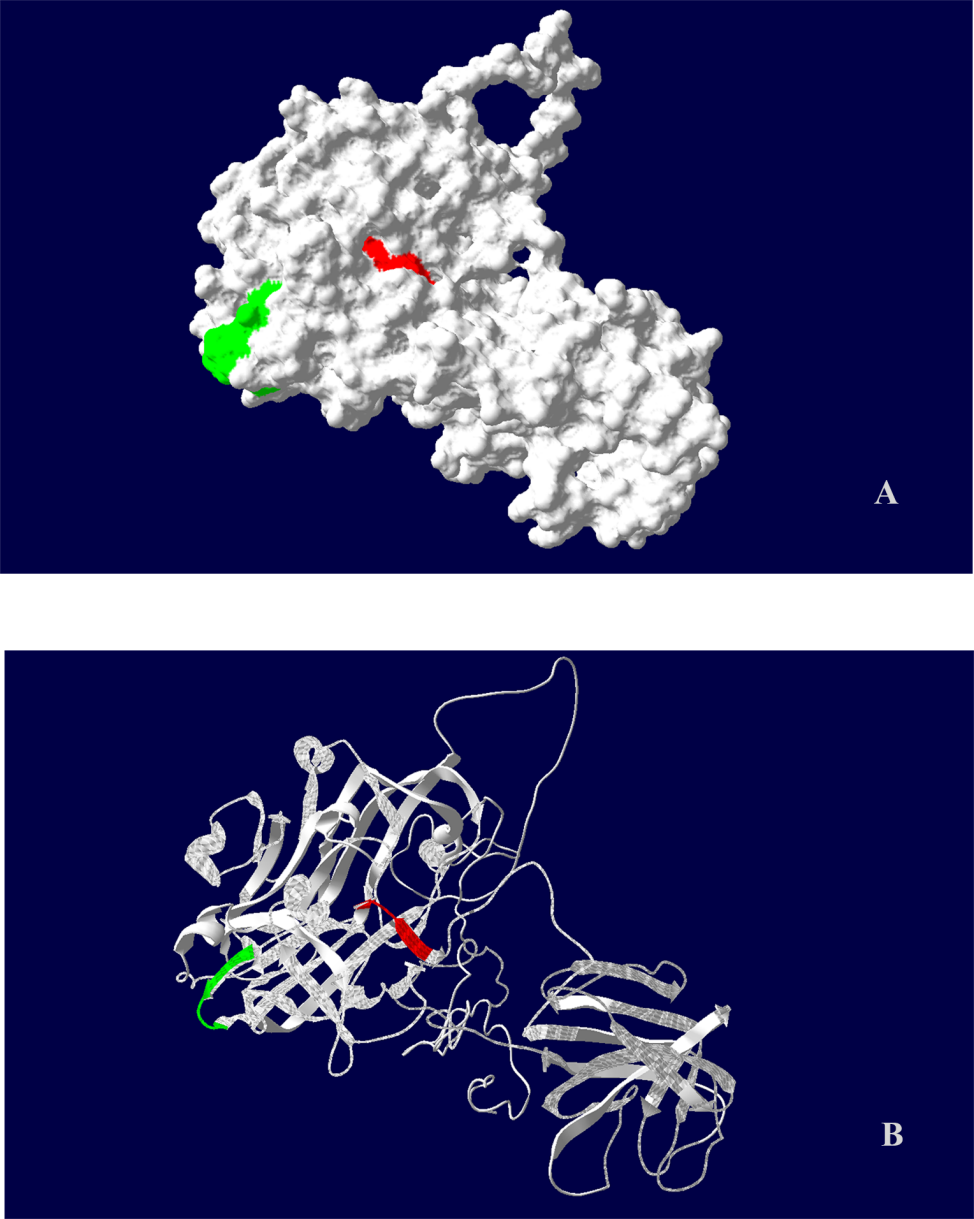
Predicted molecular model of genotype 4 HEV ORF2. (A) Molecular surface pattern. (B) Cartoon pattern. IVIPHD motif indicated in green and YDNQH indicated in red.

A structural model of ORF2 protein was generated to predict the spatial location of the two suspected epitopes. Modeling analysis indicated that G438IVIPHD444 was located in an outer region protruding from the protein molecule, and Y457DNQH461 was located in a groove (Fig 5A). Both suspected epitopes were located in a random coil distributed between two β-sheets (Fig 5B).

### Affinity of the synthetic peptides

**Fig 6:**
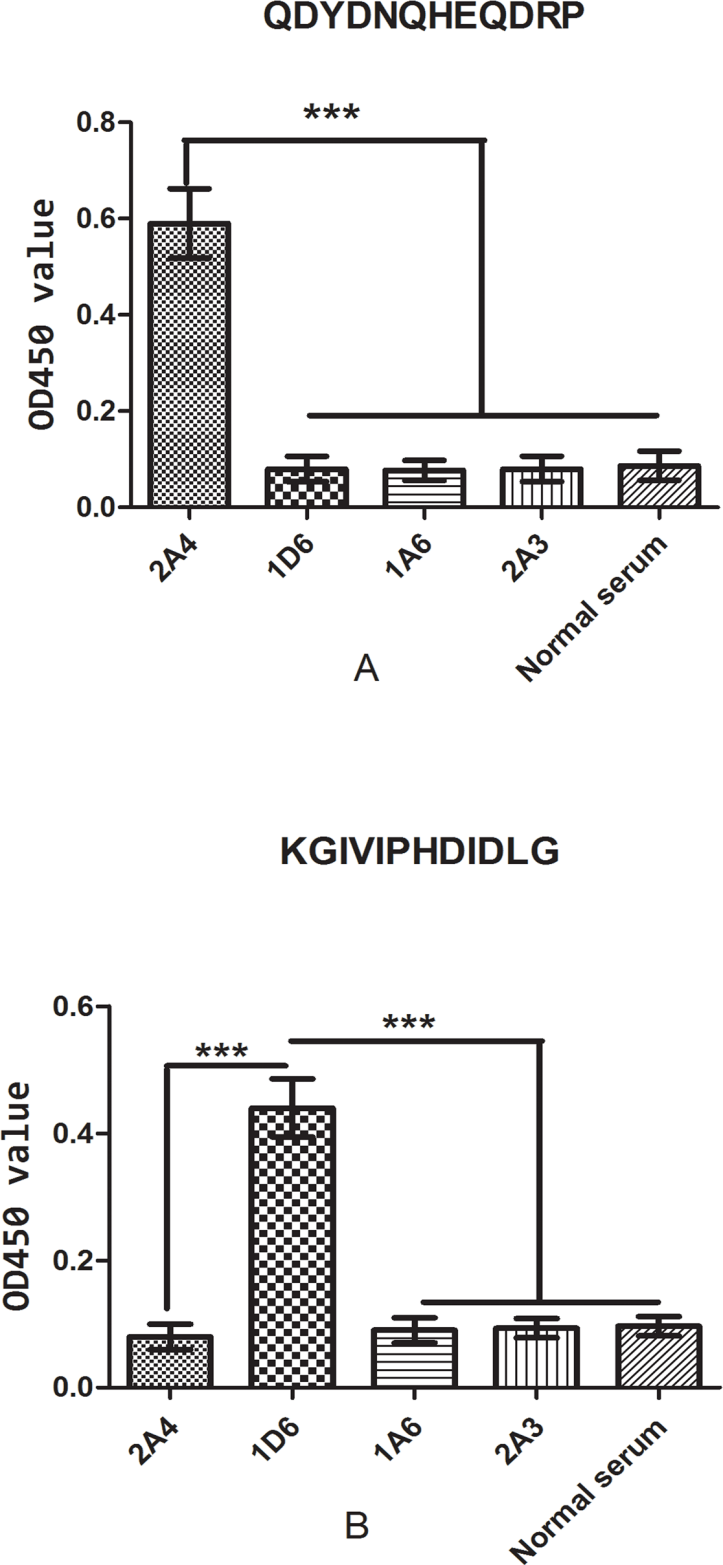
Binding affinities of synthetic peptides to prepared mAbs. (A) ELISA of peptide QDYDNQHEQDRP. (B) ELISA of peptide KGIVIPHDIDLG. OD_450_ values are shown on the *y* axis. The names of the prepared mAbs and control are shown on the *x* axis. ***P < 0.001.

In view of the aforementioned finding, two peptides, QDYDNQHEQDRP and KGIVIPHDIDLG, were synthesized to identify the binding activity of the two suspected epitopes. The two peptide sequences corresponded to the Q455DYDNQHEQDRP466 and K437GIVIPHDIDLG448 domains of HEV genotype 4 ORF2 protein, which contained the two suspected epitopes. The synthetic peptides QDYDNQHEQDRP and KGIVIPHDIDLG showed specific affinities to their corresponding mAbs 2A4 and 1D6 as demonstrated by ELISA, in contrast to the other three mAbs and the negative control (*P*<0.001) (Fig 6).

### Preliminary evaluation of peptide-based ELISA

The two peptides QDYDNQHEQDRP and KGIVIPHDIDLG conjugated to bovine serum albumin at their N-terminus were coated on ELISA plate wells. Serum samples identified by commercial ELISA were selected to evaluate the peptide-based ELISA method. The results of the peptide-based ELISA method were consistent with those of the commercial ELISA kit for detecting negative samples. However, only 83 out of 100 positive serum samples confirmed by commercial kit were identified by QDYDNQHEQDRP-based ELISA, and 87 out of 100 by KGIVIPHDIDLG-based ELISA. The total coincidence rates of these two peptide-based ELISA methods with commercial kits were 90.9% and 93.0%, respectively (Table 5). These results suggest that these two peptide-based ELISA methods had good specificity but low sensitivity compared with the commercial method. Nevertheless, these two peptides including the newly indentified epitopes of swine HEV ORF2 protein could sever as potential candidate targets for diagnosis in further research.

**Table 5:** Compliance between commercial ELISA kit and ORF2 peptide-based ELISA.

	Positive no.	Negative no.	Total coincidence rate	
Commercial ELISA kit	100	86		NA
QDYDNQHEQDRP-based ELISA	83 (17 negative)	86		90.9%
KGIVIPHDIDLG-based ELISA	87 (13 negative)	86		93.0%

## Discussion

In the present study, we prepared four mAbs against HEV genotype 4 ORF2 protein and identified two linear B cell epitopes located in the regions of G438IVIPHD444 and Y457DNQH461 in ORF2. Previous studies revealed critical epitopes distributed in the ORF2 C-terminal, which may be masked by larger recombinant expression products of ORF2 protein [16]. We therefore cloned a truncated gene from nucleotides 1240–2022 of HEV genotype 4 ORF2, and inserted it into the pET32a vector to express a recombinant truncated fragment of ORF2 (amino acids 414–674). This His-tag fused fragment was then expressed and purified as an immunogen to prepare the mAbs, with His-tagged recombinant protein used to exclude false positives. We subsequently identified four mAbs with a specific reaction to the ORF2 fragment, all of which could be used to detect ORF2 expression at the subgenomic level, as confirmed by IFA in BHK cells transfected with pcDNA3.1-ORF2 plasmids.

Various types of polypeptides and truncated recombinant proteins have been shown to include immunodominant epitopes of ORF2 protein and could thus be used as reagents to detect anti-HEV IgM or IgG [17,18,19]. However, the precise locations of the epitopes in the ORF2 protein remain unclear. We therefore used the four purified mAbs as immobilized targets to screen the ORF2 epitopes by phage display, which is an efficient and widely used tool for epitope mapping of target proteins [12,20]. After four rounds of biopanning, we selected phage clones corresponding to the respective mAbs and amplified their exogenous sequences using specific primers. Sequencing and alignment analysis indicated strong homology among the 12-mer peptide sequences displayed on the phages to mAb 2A4 and 1A6. In addition, one consensus peptide sequence displayed on the phages to mAb 1D6 also showed high homology to peptide sequences displayed on the phage to mAb 2A4.

We previously expressed two recombinant HEV genotype 4 ORF2 fragments, ORF2-1 (amino acids 414–571) and ORF2-2 (amino acids 524–674), and we then used these and the four mAbs prepared in the present study to preliminarily identify the epitope distributions of ORF2 by western blotting [21]. All four mAbs interacted specifically with ORF2-1 but not ORF2-2, suggesting that the epitopes corresponding to these mAbs were located in the region of amino acids 414–523.

We compared all the peptide sequences with the HEV genotype 4 ORF2 protein and identified a series of consecutive amino acids displayed on the phages corresponding to mAbs 2A4, 1A6, and 1D6, which were analogous to the Y457DNQH461 and G438IVIPHD444 motifs. Some of the peptide sequences, notably sequences C1 and A4, contained sequences that were completely consistent with these two motifs, while other homologous sequences contained some amino acid mutations, including Y to F, Q to N, V to L, and I to V substitutions. L, V, and I are aliphatic, while F and Y are aromatic, and Q and N are amides. Given that amino acids in the same series have similar structures and characteristics, we conclude that the amino acid sequences YD#QH and I#IP#D represented the core sites of Y457DNQH461 and G438IVIPHD444, respectively. According to the binding activity analysis of the phages to mAbs 2A4 and 1A6, we speculated that the Y457DNQH461 and G438IVIPHD444 motifs may represent two epitopes of HEV genotype 4 ORF2 protein. The current results thus provide more precise locations for epitope regions covered in earlier studies [18,22,23].

Furthermore, we examined the conservation of the two suspected epitopes among a panel of ORF2 protein sequences including genotypes 1, 2, 3, 4, and avian HEV isolates, and demonstrated high sequence homology in these two regions among genotypes 1, 2, 3, and 4 isolates, except for a minor amino acid mutation in a few isolates. We also detected a D to G mutation in the avian isolate compared with the Y457DNQH461 motif of HEV genotype 4, and a two-amino acid AI to MV mutation compared with the G438IVIPHD444 motif. However, we concluded that these mutations might not have a major influence on this domain, given that I and V are both branched chain amino acids, which also include L. Overall, these two suspected linear epitopes may thus be universal to genotypes 1, 2, 3, 4, and avian HEVs. Two mAbs against avian HEV ORF2 protein were previously used to screen its epitopes, and an I/VPHD motif was identified as a novel linear epitope in the capsid (ORF2) protein of avian HEV, which is speculated to be common to both human and swine HEVs according to sequence alignment and ELISA analysis [24]. This result was confirmed in the present study with regard to genotype 4, confirming that the I/VPHD domain is a universal linear epitope in swine, human, and avian HEV isolates.

Structural prediction indicated that the two suspected epitopes were located in a random coil between two β-sheets. The random coil structure could promote the activity of molecular and β-sheets to maintain the stability of large fragments, and would thus allow the activity of the two suspected epitopes. In addition, the G438IVIPHD444 motif was located in an outer region and was thus potentially a more suitable epitope than Y457DNQH461, which was located in a groove.

We analyzed the binding activity of the two suspected epitopes by synthesizing two 12-mer peptides Q455DYDNQHEQDRP466 and K437GIVIPHDIDLG448 of ORF2 protein, which contained Y457DNQH461 and G438IVIPHD444, respectively, and confirmed the specificity of these two synthetic peptides to their corresponding mAbs by ELISA. Peptide-based immunoassays are widely used for diagnostic tests [25,26,27], and we therefore developed a peptide-based ELISA to detect HEV in serum using these two synthetic peptides as antigens. Preliminary results indicated that both peptides exhibited similar antigenic activities, and the peptide-based ELISAs revealed good specificity but low sensitivity compared with the commercial method. These results suggest that these two peptides including the newly identified epitopes could serve as potential candidate targets for diagnostic tests, though further studies are needed to optimize the peptide-based ELISAs.

In conclusion, we prepared four mAbs against the ORF2 protein of HEV genotype 4, which could be used to detect ORF2 protein using subgenomic expression strategies. Furthermore, we identified the epitopes G438IVIPHD444 and Y457DNQH461 corresponding to two of these mAbs using phage display biopanning technology. Both epitopes were speculated to be universal to genotypes 1, 2, 3, 4, and avian HEVs. In addition, a peptide-based ELISA using two 12-mer fragments of ORF2 protein including these two epitopes was developed to detect HEV, and demonstrated good specificity but low sensitivity compared with the commercial method. These newly characterized epitopes may thus represent potential candidate targets for diagnostic tests. Further research is required to optimize this peptide-based ELISA method, and to confirm the universality of these epitopes in genotypes 1, 2, 3, and avian HEVs using more systematic experiments.

## Supporting information

S1 Fig HEV ORF2 protein expression in BHK cells transfected with pcDNA3.1-ORF2 by mAbs 1D6, 1A6, and 2A3. BHK cells transfected with pcDNA3.1 were designated as the control.
(TIF)

S1 Table Enrichment efficacy of biopanning for ORF2 mAbs. (DOC)
