# Supplementary figures and images for: Identification and characterization of two linear epitope motifs in hepatitis E virus ORF2 protein
Source: PLoS One. 2017 Sep 28;12(9):e0184947. doi: 10.1371/journal.pone.0184947 (PMC5619941; doi:10.1371/journal.pone.0184947)

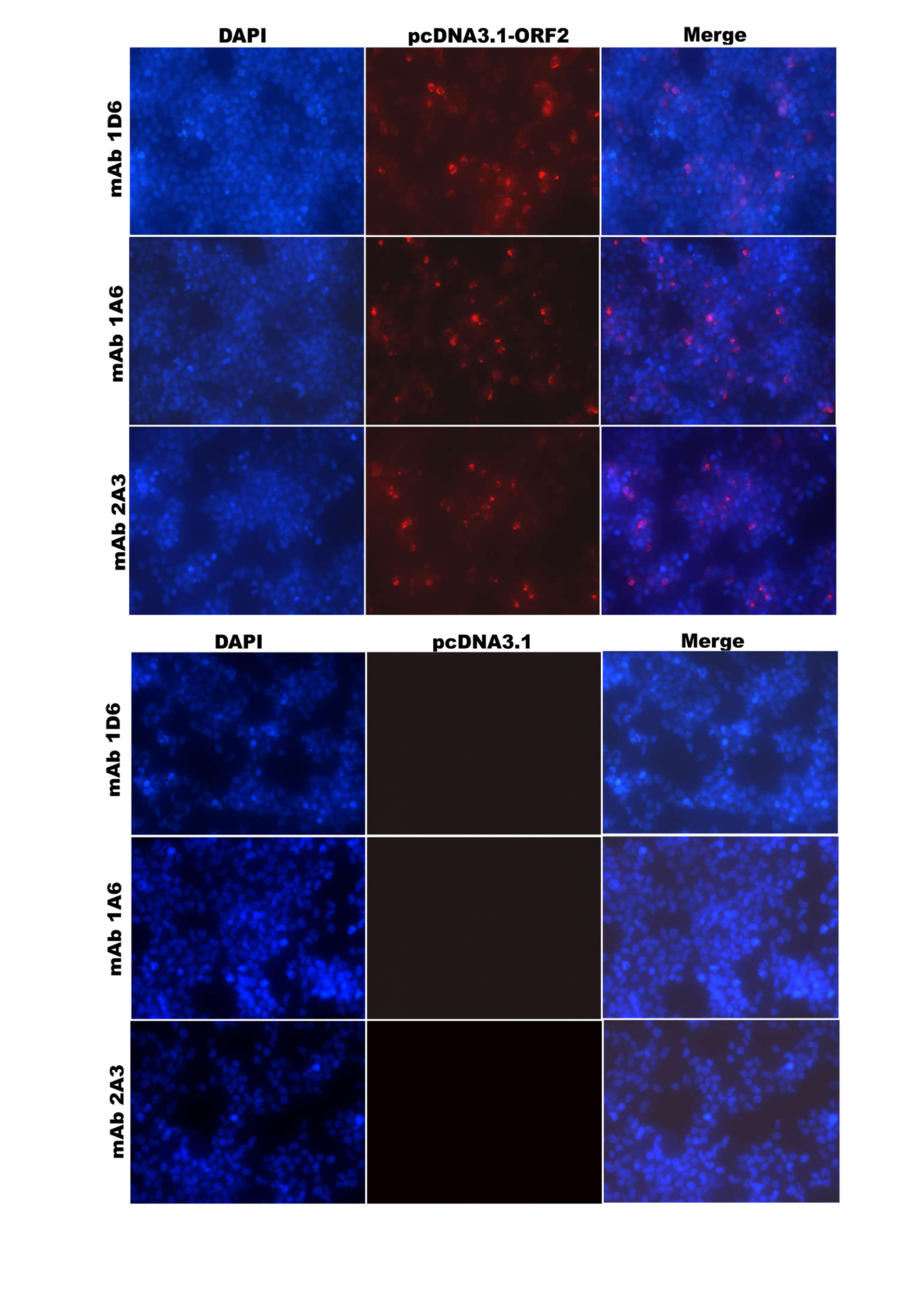

Supplement: S1 Fig — BHK cells transfected with pcDNA3.1 were designated as the control. (TIF) [file pone.0184947.s001.tif]
