# Supplementary material for: Identification and characterization of two linear epitope motifs in hepatitis E virus ORF2 protein
Source: PLoS One. 2017 Sep 28;12(9):e0184947. doi: 10.1371/journal.pone.0184947 (PMC5619941; doi:10.1371/journal.pone.0184947)
Supplement: S1 Table — (DOC) [file pone.0184947.s002.doc]

| **mAb** | **Round of biopanning** | **Input**  **(pfu)** | **Output**  **(pfu)** | **Output/ Input** |
| --- | --- | --- | --- | --- |
| 2A4 | 1st | 4×10^10^ | 1.2×10^4^ | 3.0×10^-7^ |
|  | 2nd | 1×10^11^ | 2.4×10^5^ | 2.4×10^-6^ |
|  | 3rd | 1×10^11^ | 3.2×10^6^ | 3.2×10^-5^ |
|  | 4th | 1×10^11^ | 6.1×10^7^ | 6.7×10^-4^ |
| 1D6 | 1st | 4×10^10^ | 1.3×10^4^ | 3.25×10^-7^ |
|  | 2nd | 1×10^11^ | 7.1×10^5^ | 7.1×10^-6^ |
|  | 3rd | 1×10^11^ | 2.6×10^7^ | 2.6×10^-4^ |
|  | 4th | 1×10^11^ | 1.3×10^8^ | 1.3×10^-3^ |
| 1A6 | 1st | 4×10^10^ | 1.2×10^4^ | 3.0×10^-7^ |
|  | 2nd | 1×10^11^ | 6.3×10^5^ | 6.3×10^-6^ |
|  | 3rd | 1×10^11^ | 9.1×10^6^ | 9.1×10^-5^ |
|  | 4th | 1×10^11^ | 8.1×10^8^ | 8.1×10^-3^ |
| 2A3 | 1st | 4×10^10^ | 1.6×10^4^ | 4.0×10^-7^ |
|  | 2nd | 1×10^11^ | 3.3×10^5^ | 3.3×10^-6^ |
|  | 3rd | 1×10^11^ | 2.7×10^6^ | 2.7×10^-5^ |
|  | 4th | 1×10^11^ | 7.1×10^8^ | 7.1×10^-3^ |
